# Collaborative Use of Sensor Networks and Cyberinfrastructure to Understand Complex Ecosystem Interactions in a Tropical Rainforest: Challenges and Lessons Learned

**DOI:** 10.3390/s23229081

**Published:** 2023-11-09

**Authors:** Philip W. Rundel, Thomas C. Harmon, Angel S. Fernandez-Bou, Michael F. Allen

**Affiliations:** 1 Department of Ecology and Evolutionary Biology, University of California, Los Angeles, CA 90095, USA; 2 Sierra Nevada Research Institute, Department of Civil and Environmental Engineering, University of California, Merced, CA 95343, USA; tharmon@ucmerced.edu (T.C.H.); afernandezbou@ucsusa.org (A.S.F.-B.); 3 Climate & Energy Program, Union of Concerned Scientists, 500 12th St., Suite 340, Oakland, CA 94607, USA; 4 Center for Conservation Biology, Department of Microbiology and Plant Pathology, University of California, Riverside, CA 92507, USA; mallen@ucr.edu

**Keywords:** tropical forest, global change, carbon cycle, eddy covariance, soil fluxes, leaf-cutter ants, research challenges

## Abstract

Collaborations between ecosystem ecologists and engineers have led to impressive progress in developing complex models of biogeochemical fluxes in response to global climate change. Ecology and engineering iteratively inform and transform each other in these efforts. Nested data streams from local sources, adjacent networks, and remote sensing sources together magnify the capacity of ecosystem ecologists to observe systems in near real-time and address questions at temporal and spatial scales that were previously unobtainable. We describe our research experiences working in a Costa Rican rainforest ecosystem with the challenges presented by constant high humidity, 4300 mm of annual rainfall, flooding, small invertebrates entering the tiniest openings, stinging insects, and venomous snakes. Over the past two decades, we faced multiple challenges and learned from our mistakes to develop a broad program of ecosystem research at multiple levels of integration. This program involved integrated networks of diverse sensors on a series of canopy towers linked to multiple belowground soil sensor arrays that could transport sensor data streams from the forest directly to an off-site location via a fiber optic cable. In our commentary, we highlight three components of our work: (1) the eddy flux measurements using canopy towers; (2) the soil sensor arrays for measuring the spatial and temporal patterns of CO_2_ and O_2_ fluxes at the soil–atmosphere interface; and (3) focused investigations of the ecosystem impact of leaf-cutter ants as “ecosystem engineers” on carbon fluxes.

## 1. Introduction

Sensor networks that take advantage of the expanded modalities for sensing capacity offer exciting advances in the design of powerful sensing arrays with new applications for ecological and ecosystem research. This progress has allowed for a broadening of the boundaries of ecosystem and ecological research thanks, in no small part, to the development of interfaces between environmental science, engineering, and informational technology. Ongoing advances in understanding the complexities of ecosystem functions and, importantly, regional and global models for biogeochemical fluxes have been spurred by the development of new sensor technologies, increased connectivity afforded by the Internet to transmit and share data, reduced size and weight of sensors, and improved reliability of environmental sensing hardware under extreme environmental conditions. Intelligent arrays of sensor networks are emerging as fundamental tools for addressing complex questions of ecosystem structure and stability in a world with increasing impacts of global change. The key to these advances has been the innovative application of cyber infrastructure linking advanced instruments, computing systems, data storage systems, and repositories [1,2]. These advances often have come, however, with significant challenges, particularly under extreme environmental conditions.

Sensor networks coupled with associated cyberinfrastructure can provide a powerful combination of distributed sensing capacity, internet and satellite communication, and computational tools that lend themselves to countless applications in ecological research [3,4]. Moreover, new designs for sensor networks allow for the observation of system fluxes in near time and real time based on incoming data from local sources, nested or adjacent networks, and remote sensing data streams. These advances are providing a new and better understanding of dynamic ecological systems by revealing previously unobservable phenomena and by enhancing the potential for the development of second-generation ecological questions that we have not yet addressed [4]. 

Additionally, new observational and experimental advancements are available for the first time, enabling scientists to address challenging questions using enhanced spatial and temporal sampling. These powerful innovations can deliver: (1) full four-dimensional access to the environment with long-lived, continuously operating, stationary and mobile sensor nodes suspended in the forest canopy; (2) self-organized, standards-based, broadband wireless networks and software systems, permitting broadband access to sensor nodes; and (3) the integration of remote sensor data sources with existing GIS database assets to provide a comprehensive, continuous, online portal for access to embedded networked sensor data.

## 2. Tropical Rainforests as Drivers of Global Change

Although tropical forests cover only about 7% of the land area of the world, they store one quarter of global terrestrial carbon and account for approx. one-third of global net primary productivity. Importantly, humid tropical forests in particular play a crucial role in regulating regional and global climate dynamics [5]. Understanding their responses to global change is crucial for predicting biogeochemical global carbon and hydrological cycling. However, the responses of tropical forests to environmental changes are complex and poorly understood, and important knowledge gaps exist in the development of models for predicting tropical forest dynamics. There is abundant evidence that global changes are altering the physical and chemical environment in which tropical trees grow and that these changes have been occurring for decades across large areas [6]. However, many of these predictions are based on results from leaf-level studies. How tropical forests respond and feed back to climate change remains poorly understood at the ecosystem level [7]. Four critical research questions have been identified as priority needs for policy makers, conservationists, and ecosystem ecologists. (1) How are dynamic changes in land use altering the biogeochemical processes in tropical forest ecosystems? (2) How does the spatial variability in tropical forests impact global change? (3) Are there emergent trends in the ecosystem responses of tropical forests to global change? (4) What are the key research and management priorities for tropical forest ecosystems in the context of global change? [5,6,7,8,9]. These challenges provide critical questions for research and the areas where the innovative use of sensor arrays can be useful. We briefly describe three areas of sensor research for our group, which involve linked, spatial, and temporal scales of impact. A major focus here is not to present the research results from our studies but rather offer a commentary on the lessons learned and the environmental challenges we faced in working with sensors and sensor arrays in a wet tropical forest environment.

## 3. La Selva Biological Station, Costa Rica

Our work involved a mix of traditional and new modalities of networked sensors. In this commentary, we report on three components of our ongoing research on the biogeochemical cycles of carbon in tropical forest ecosystems, each operating at a different spatial and temporal scale. The first component involves the use of instrumented canopy towers for eddy flux measurements. The second describes the development of novel systems of soil sensor arrays with both environmental monitoring and automated imaging for root and mycorrhizal fungal growth. The third area of research takes a downscaled approach to quantify the role of leaf-cutter ants whose life history and behavior as ecosystem engineers influence the biogeochemical cycles in Neotropical forests. We present brief overviews of these three areas of research, the multiple challenges in the installation, operation, and maintenance of the sensors and networked sensor arrays in a wet tropical forest, and the environmental conditions of high humidity, torrential rains, ubiquitous annoying insects, venomous snakes, and dynamic forest structures with frequent tree falls.

**Figure 1:**
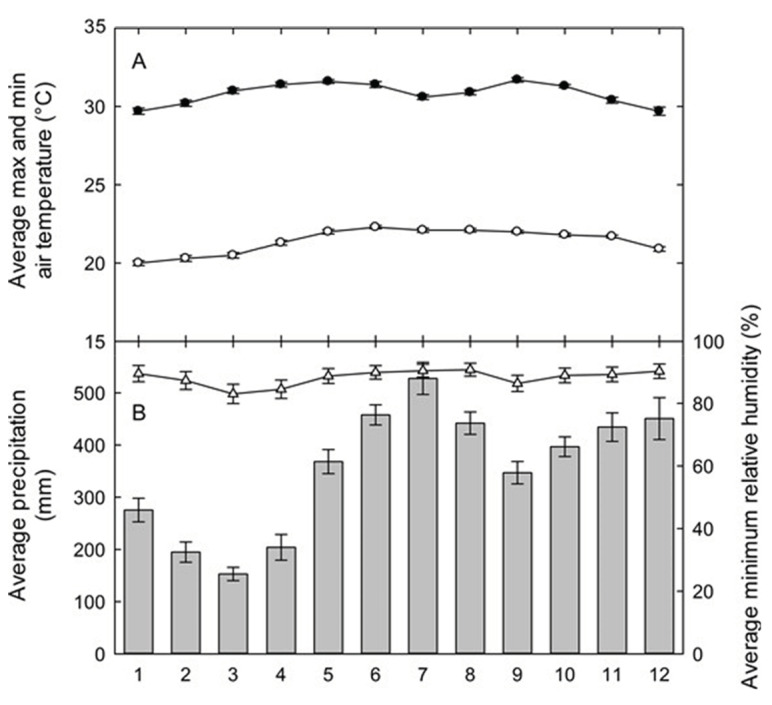
Seasonal climate means for the La Selva Biological Station: (**A**) The mean daily maximum (closed circles) and minimum (open circles) air temperatures, and (**B**) the total monthly precipitation (vertical bars) and mean daily minimum relative humidity (open triangles) for the La Selva Biological Station, Costa Rica. The values are the means ± standard errors for each month for the data collected hourly from 1957 to 2003 for the precipitation, from 1982 to 2003 for the temperature, and from 1992 to 2003 for the relative humidity. Graph courtesy of Eric Graham.

Our research efforts for the last decade have centered on tropical rainforests at the La Selva Biological Station, a 1600-hectare reserve of premontane wet forest in the Caribbean lowlands of northwestern Costa Rica. This field site is extremely wet, averaging 4244 mm of annual precipitation (1958–2004), with a mean monthly rainfall above 300 mm from May through December (Figure 1). The peaks of monthly rainfall above 400 mm typically are present in June–August and November–December, and a drier period occurs from January through April. Even in the driest period in February and March, however, precipitation averages above 190 mm each month. There is little seasonal change in the mean monthly maximum and minimum temperatures. The developed area of La Selva includes more than 20 buildings with housing and dining for groups or individuals, air-conditioned laboratories and research buildings, an ambient temperature lab, shade house growth facilities, and a well-equipped GIS lab serving the research and academic community. The GIS database contains over 1000 coverages, including aerial photographs, satellite images (LANDSAT, IKONOS, QuickBird), optical scans (multispectral and hyperspectral), and active sensor images (RADAR, LiDAR) of La Selva and its broader watershed for several time periods over the past 45 years. La Selva follows the protocols recommended by the World Meteorological Organization (WMO) for sensor handling, data collection and management, and QA/QC [10].

**Figure 2:**
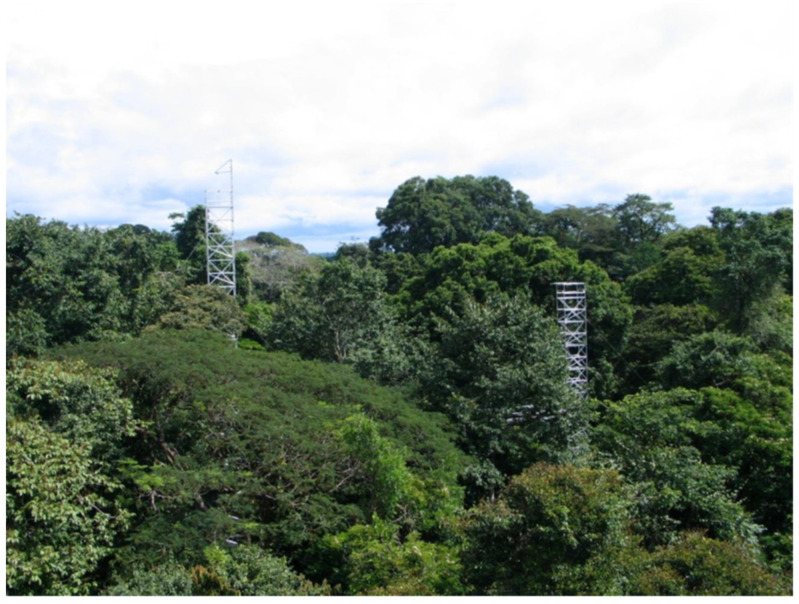
Two of the instrumented La Selva canopy towers reaching above the forest canopy. La Selva Biological Station.

**Figure 3:**
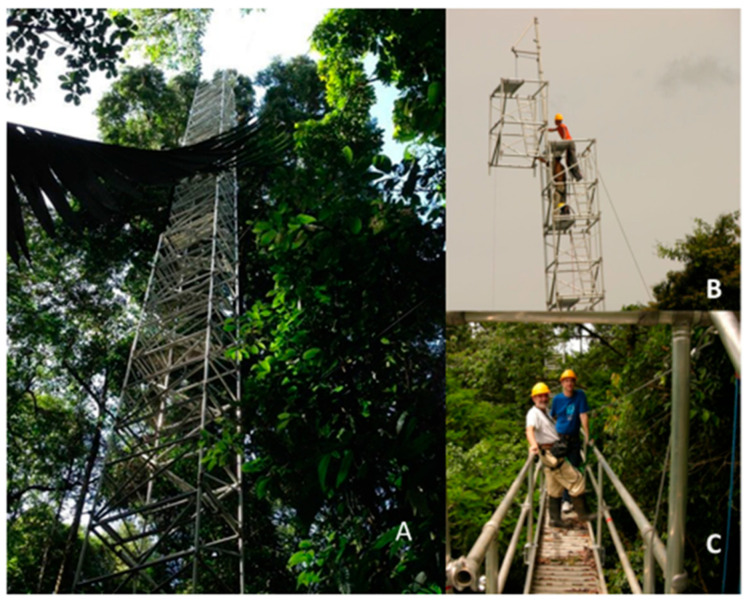
La Selva Canopy Tower Network. (**A**) Tower from below; (**B**) installation of instrumentation for eddy covariance measurements at the top of the tower; (**C**) canopy walkway between the two tours at 25 m above the forest floor. La Selva Biological Station.

The La Selva Canopy Tower Network, constructed in 2008 with funding from the U.S. National Science Foundation, consists of a network of canopy towers in an old-growth forest (Figure 2). They are linked together by both fiber optic and wireless networks, with fiber optic and electrical connections extending for 1 km to the laboratory buildings. In their original architecture, the scaffolding towers were arranged in a triangle with heights of 34 m, 42 m, and 51 m, with the two shorter towers connected by a 25-m walkway through the canopy to provide expanded access to the interior tree crowns. The modular form of these towers allows for relative ease in reconfiguring the height (Figure 3). 

Underground conduits provide a direct connection of 600-V power and fiber optic cables from the towers to the laboratory area located 1 km away. An air-conditioned hut is available near the base of one of the towers to protect the instrumentation requiring temperature and humidity control. Three additional towers consist of triangular radio (climb-up) towers that provide canopy access points for audio, sensor, or image recording. The tower footprint offers electricity at both 120 V and 240 V, wireless internet, and cloud access points. 

The bandwidth from the towers to the station is ample (at least 6 mbps) and tied to the station’s bandwidth out of the station’s 40 mbps capacity. High bandwidth users (e.g., high-frequency image files) also have the option of storing their data on station-based servers and collecting data from the station at low bandwidth use periods or via media transfer. Internet access has improved in the past 10 years from a 50-kbps telephone line to 40 mbps, including a dedicated line between La Selva and the OTS central offices located on the research campus of the Universidad de Costa Rica in San José. Internally, the system is a combination of optical fiber, UTP, and wireless connections.

The data streams from the Canopy Tower Network provide continuous data on ecosystem fluxes and concurrent detailed canopy and soil environmental conditions as needed for a broad range of data analyses, syntheses, and modeling efforts (e.g., [11,12,13,14]). The profile system includes sensors for light (PAR), air temperature, and humidity at four canopy heights (top, upper, middle, and lower canopy). Additional biomet sensors provide data on the canopy profiles of light and atmospheric conditions that are useful for flux data analysis and as the input for modeling leaf fluxes using multi-layer ecosystem models. A strong focus on carbon cycling and global change has developed around the tower facility (e.g., [15,16,17,18,19]).

## 4. Eddy Covariance Research

One the best examples of successful efforts to apply new technologies to tropical forest research came two decades ago with the installation of eddy flux towers within the Large-scale Biosphere–Atmosphere Experiment (LBA) in Amazonia, an international research program involving collaborations between hundreds of scientists from Brazil, the United States, and other countries. Patterned after a previous successful program of flux studies on boreal ecosystems in Canada, the LBA was motivated by the need for improved observations of carbon, evapotranspiration, and sensible heat fluxes in the tropics to understand how changes in land use and climate might affect the sustainability of the biological and physical functions of Amazonia, as well as their subsequent influence on the global climate system. The eddy covariance method provides a means to directly and non-intrusively estimate the vertical transport of CO_2_ and other greenhouse gases through and out of the forest canopy. These data, coupled with the appropriate sensors, can provide invaluable data to improve our understanding of carbon cycles, energy fluxes, and other atmospheric controls. 

The measurements of the net CO_2_ flux between terrestrial ecosystems and the atmosphere using eddy covariance techniques have been fundamental to the development and application of global and regional carbon source-sink patterns and predictions of the future terrestrial carbon balance [11,13,20,21]. The LBA studies suggest that Amazonia has been, on average, nearly neutral with respect to carbon over the last decade, albeit a small net source during ENSO events. Despite several decades of eddy flux measurements at multiple sites across the Amazon Basin, regional biogeophysical models have had difficulties in reproducing the seasonal pattern of the net ecosystem exchange of carbon dioxide. The eddy covariance measurements of NEE were taken in an old-growth forest.

The global network of eddy covariance towers (FLUXNET) currently consists of more than 500 active sites, but humid tropical forests are underrepresented [11]. La Selva has been a flux site since 1998, and past research has identified a large sensitivity of nighttime temperature fluxes. The daytime net ecosystem fluxes were largely controlled by solar radiation rather than by evaporative demand. The dry season vapor pressure deficit was identified as a main control for forest growth on interannual timescales [22,23,24,25]. The eddy covariance research over the last two decades at La Selva has had challenges in maintaining continuous measurements across the seasons and has further highlighted the challenges of extrapolating data to broader forest areas. 

The eddy covariance analyses carried out from 1984–2000 at La Selva found that the canopy tree growth in the old-growth forest varied >two-fold among years. The trees’ annual diameter increments in this 16-yr period were negatively correlated with the annual means of the daily minimum temperatures [22]. The tree growth variations were also negatively covaried with the net carbon exchange, as inferred by an inverse tracer–transport model. The measurements during the 1997–1998 El Niño year found significant reductions in tree growth, and large inferred tropical releases of CO_2_ to the atmosphere occurred. These and other recent findings are consistent with the decreased net primary production in tropical forests in warm years. 

Previously, the walk-up towers at La Selva were similar in height to the scattered emergent tree crowns. With a new 60-m tower, the eddy covariance system will be located sufficiently above the mean canopy height to minimize any potential interference of the tallest trees with the eddy covariance measurements [21]. The canopy profile system will soon be monitoring light (PAR) and CO_2_–H_2_O concentrations at 5-m height intervals from 0 m to 50 m. The data from the profile system will be used to calculate the canopy storage of CO_2_, which is an important component of the ecosystem carbon flux budget. Tropical forests often experience extended periods of very calm air, and under these conditions CO_2_ can accumulate inside the canopy air space. This has presented a serious challenge in the past at La Selva. However, the new CO_2_ profile system should provide novel insights into the role of carbon storage in creating biases in the ecosystem carbon flux budgets measured by above-canopy eddy covariance systems. 

A team of researchers from UCLA is in the early stages of an NSF project linked with the eddy covariance towers to carry out an innovative carbonyl sulfide project (NSF funded, Ulli Seibt, UCLA). The goal of the project is to quantify ecosystem photosynthesis and respiration for the tropical rainforest at La Selva and link these fluxes to environmental variations. The researchers will measure ecosystem fluxes of carbonyl sulfide (COS), a novel tracer of photosynthesis, and obtain estimates of ecosystem photosynthesis from the COS fluxes. The data will be used in ecosystem modeling to better constrain the model simulations of tropical photosynthesis. An eddy covariance inlet and automated chambers will be installed on the height extended tower and will be connected via tubing to a high-precision COS laser spectrometer housed in the air-conditioned instrument shed. The shed is critical for high-quality measurements since the laser spectrometer is sensitive to temperature fluctuations.

## 5. Soil Sensor Arrays

**Figure 4:**
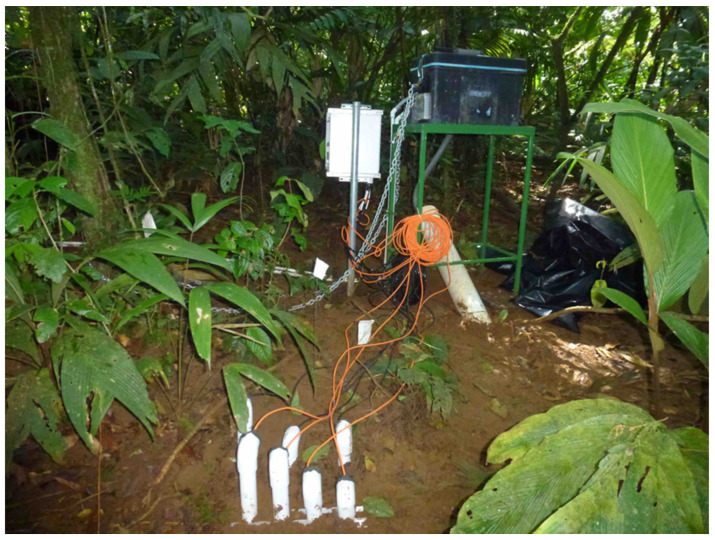
Soil Ecosystem Observatory at the La Selva. The white box holds a Campbell data logger for recording the sensor data inputs at 5 min intervals. Attached to the data logger are the wiring routed through PVC tubes into the soil with buried sensors to measure the temperature and moisture (Campbell Scientific, Logan, Utah), CO_2_ (Vaisala, Helsinki, Finland), and O_2_ at four depths, 2, 8, 16, and 50 cm (see details in text [29]). The black box houses the PC for recording the images from the automated minirhizotron (AMR). The cable runs from the computer to the AMR within the PVC tube at a 45° angle and a meter in length (70 cm depth).

Tropical rainforest soils receive large inputs of carbon from the atmosphere through photosynthesis. However, organic matter decomposition is also very high as moisture and temperature are optimal for microbial growth and turnover, releasing that carbon back to the atmosphere. The primary limitation is presumed to be nutrients due to high leaching rates and saturated moisture levels that restrict O_2_ for aerobic microbial respiration. Nevertheless, when carefully examined, the tropical rainforest soils at L Selva have been found to have high root, fungal, and bacterial standing crops [26]. Further, organic carbon may well be an important sink because of the interconnected mycorrhizal plant–fungal–soil pathways [27]. Therefore, establishing the relative significance of the pathways of carbon flow is an important step (Figure 4).

A key element of our sensor studies has been the development of a soil monitoring system installed near the base of the eddy covariance towers. The system consists of three instrumented soil profiles, each with sensors for soil CO_2_ and O_2_, soil temperature, and soil moisture at four depths (surface, 5–10, 15–20, 50 cm) totaling 12 sets of sensors. Additional surface sensors for the soil temperature and moisture were placed to generate spatial information about the surface soil conditions and select the representative areas for long-term monitoring. The sensor profiles provide data on the soil dynamics at a level of detail not typically present at most flux tower sites. In our measurements to date, we have found that soil respiration is a major component of the ecosystem carbon flux, which is estimated to comprise approx. half of the total ecosystem respiration. As the new eddy covariance towers become operational, the soil flux data will serve as an important check on the carbon flux budget.

The CO_2_ sensors consisted of a remote Vaisala probe (GMT220 transmitter and GMP221 probe, 0–10% CO_2_ range) and the O_2_ sensors (Apogee model SO-110). The sensors were installed inside a sealed top of polyvinyl chloride (PVC) tubing and exposed end sealed with a GORE-TEX lining located at the appropriate depth. The temperature and moisture readings were undertaken using Campbell Scientific CS650 sensors (Logan, UT, USA). All the sensors were connected to the same data logger for synchronized data. The readings were recorded at 5-min intervals and could be analyzed independently or averaged using 24-h analyses.

**Figure 5:**
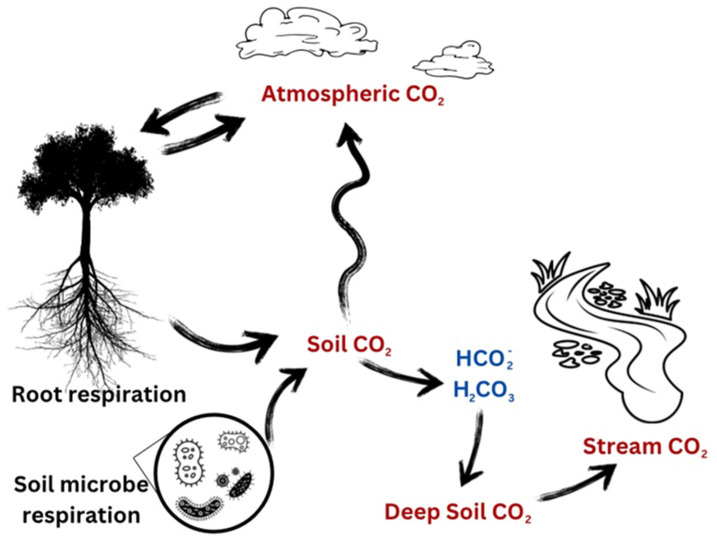
Carbon cycling model showing the carbon inputs and outputs to the atmosphere and soil.

A challenging goal for soil ecosystem research has been the quantification of root and microbial dynamics and coupling those measurements with sensor data for the soil temperature, water content, and CO_2_ and O_2_. Our future intent is to collect quantitative sufficient data to model the CO_2_ flux using HYDRUS or other existing models to utilize our soil sensor data (Figure 5). Our initial studies of soil CO_2_ fluxes have been made within the footprint of the past eddy covariance estimates [12] to better understand how soil CO_2_ behaves in this ecosystem [28,29].

The reported soil respiration values were low at La Selva, suggesting that we still have a poor understanding of the processes that regulate soil carbon dynamics in this ecosystem (Figure 4). The efflux values measured at La Selva were in the range of 3 to 4 μmol m^−2^ s^−1^ [28] in our studies. However, previous studies at La Selva have reported low rates of soil CO_2_ production of only 1 to 5 μmol m^−2^ s^−1^ [28]. In a seasonal dry tropical forest, we identified soil respiration (Rs) rates of 7 to 8 μmol m^−2^ s^−1^, and our tropical forest measurements found soil respiration rates as high as 13 μmol m^−2^ s^−1^ [29]. 

We used a gradient-flux method based on the concentrations of CO_2_ in the soil profile to estimate soil respiration (Rs) (Figure 4). In a soil context, Rs includes both the CO_2_ production through metabolism and the physical movement of the CO_2_ molecule through soil to the atmosphere. The flux-gradient Rs technology has been demonstrated using several validation applications [30]. We identified the differences in the soil CO_2_ concentrations after applying temperature and pressure corrections at multiple depths and computing the flux based on diffusion kinetics and a gas diffusivity model based on Fick’s first law for calculating the soil CO_2_ efflux. We compared the modeled fluxes to the manual measurements using above-ground chambers. This approach was complicated by the derived soil physical properties, which varied greatly across La Selva and impacted the model results.

Other parameters were found to be helpful in describing the soil processes. We used CO_2_ production (Ps) to represent the metabolic production of CO_2_, and Rs for soil ecosystem respiration. Once the concentrations at the different soil layers were known, we calculated the Ps as the inverse of the differential between the Rs values of the two layers divided by the depth. Rs integrated both the Ps and diffusion.

Biochemically derived CO_2_ comes from three primary sources: (a) autotrophic root respiration (Rr) coming from fixed CO_2_; (b) autotrophic, newly fixed CO_2_ that is respired from symbiotic microorganisms, in particular from mycorrhizal fungi (Rmf); and (c) heterotrophic CO_2_ resulting from decomposing organisms (Rh) (Figure 5). To understand the differing components, and because soil temperature in the humid tropics varies minimally, we contrasted the overall CO_2_ production with its components, Rr, Rmf, and Rh.

A key variable for modeling the soil efflux is the amount of air-filled soil pore space (θ), which is highly variable in time (wet and dry periods) and space (soil texture differences, canopy interception, microtopography), even at the experimental plot scale. The second is the tortuous particulate barriers blocking the pathway for gas molecules. The high clay content of the La Selva soils and high rainfall results in a water layer or extremely high surface water content, which restricts gas diffusion. We know little about the possible allocation of carbon to soil and its unavailability for allocation to root and microbe productivity. Is there a significant loss of carbon to CH_4_? Or is soil CO_2_ lost by dissolving the soil in water [31] and moving it out of the system by another pathway? (Figure 5). These possibilities are intriguing but challenging to address directly.

**Figure 6:**
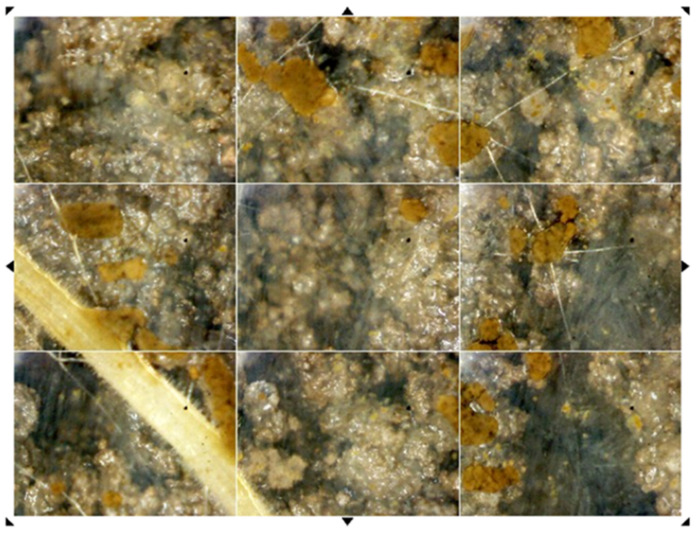
Image from the automated minirhizotron system (AMR) showing a suberizing fine root, soil aggregates, and arbuscular mycorrhizal fungal hyphal networks.

Automated minirhizotron systems (AMR) were installed adjacent to the soil sensor arrays and minirhizotrons were installed in order to image the growth of roots and mycorrhizae at rates set by the user (Figure 6). Our design utilized a Pro-Scope mounted on a sled, which moved the camera within a glass tube. The camera was connected to a computer and a 120-volt power source with a hot-wired connection for on-site image storage and wireless transfer. Each image was taken at 100×, creating an image of 3.01 mm × 2.26 mm with an average depth of field of 0.125 mm (Figure 5), and was stitched into a mosaic using RootView https://rhizosystems.com/automated-mini-rhizotron/, (accessed on 12 February 2022). The roots, fungal hyphae, soil particles, and soil invertebrates were identified and measured from the images. From these images, the roots were distinguished and the root length and standing crop from the root volume was quantified using Rootview, specifically the image recognition as described https://rhizosystems.com/image-analysis/, (accessed on 12 February 2022).

Extramatrical AM fungal hyphal lengths were found to be tightly coupled to temperature and moisture. Therefore, this study used the data from randomly chosen images from the 2010–2012 dataset to create a linear multiple regression model of the daily root respiration. The standing crop AM fungal mass (Rr) was estimated using the standing crop root biomass values of an analog species. The biomass estimates for AM fungal hyphae were determined from the length and diameter measurements and the biomass carbon determined by [32]. 

Our soil flux investigations established that there are CO_2_ pathways within tropical forest soils that remain poorly understood. One of these is a significant loss of carbon via dissolved inorganic carbon flowing into streams through impacts from animal activities, as described below for leaf-cutter ants. The root and AM fungal hyphal production and turnover increased below the ant nest footprints [33] and the nest vents created a chimney venting CO_2_ that accumulated in soil pipes created by roots [34] and fungal hypha into nests and out nest openings [34]. A second flux, which remains poorly understood, is carbon sequestration into glomalin and other organic compounds from roots, mycorrhizal fungi, and decomposed microbes. We know little about the amount of glomalin production per unit of fungal hyphal length, and a better accounting of the turnover dynamics will further our understanding of carbon sequestration in these tropical ecosystems. 

## 6. Impacts of Leaf-Cutter Ants on Biogeochemical Cycles

**Figure 7:**
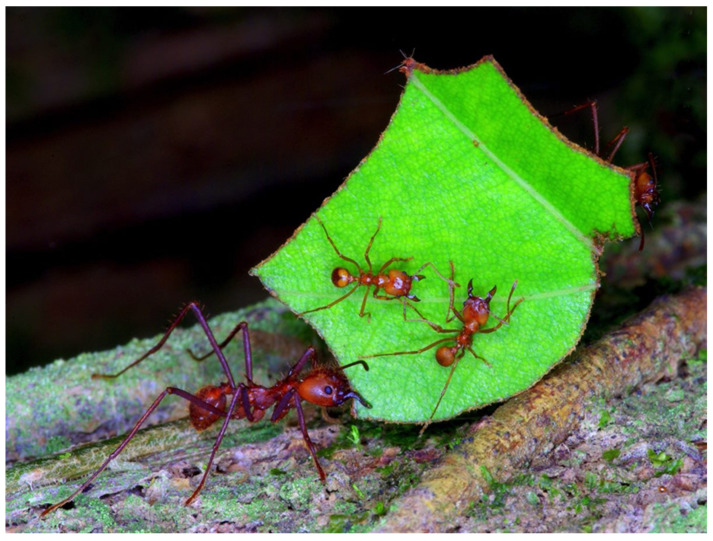
Image from the automated minirhizotron (AMR) system showing a suberizing fine root, soil aggregates, and arbuscular mycorrhizal fungal hyphal networks. Photo by Carlos de la Rosa.

Leaf-cutter ants are geographically widespread and abundant in lowland neotropical forests where they are “ecosystem engineers” with a significant effect on the ecosystem processes of carbon and nitrogen cycling (Figure 7). They account for as much as one quarter of all herbivores in these forests, and their symbionts may be responsible for significant heterogeneity in soil nitrogen resources (Figure 5). Through their herbivory, leaf-cutter ants change their environment by creating canopy gaps, transferring organic matter underground, enhancing soil aeration and turnover rates, and increasing soil nutrient availability and nitrogen fixation [35,36,37,38]. 

Leaf-cutter ants transport substantial amounts of organic material into their nests and increase the soil organic matter pool that fungi and bacteria transform into gaseous and aqueous phases. Their nests appear as mounds of excavated soil marked by numerous entrances and gas vents that lead to an intricate network of tunnels and chambers. Using the plant material they collect, leaf-cutter ants cultivate an obligate symbiotic fungus, which they consume as food. Modifying the biotic and abiotic components in their habitats and regulating the availability of resources, they influence other species.

The combined concentration of plant material with fungal and ant activity is high. This activity makes leaf-cutter nests hot spots for biogeochemical cycling [39,40]. Their nests alter the physical and chemical properties of soils, and the fungus gardens inside the nests are sites of symbiotic N_2_ fixation [38]. The roots and fungal hyphae preferentially grow inside the nests and their turnover contributes to the soil carbon pool. All these processes directly fertilize the nest soils and hypothetically promote CO_2_ efflux. As forests become increasingly fragmented across the Neotropics because of land use changes due to agriculture and grazing, leaf-cutter ants are becoming more abundant [37,40,41] and their impact on soil carbon dynamics is expected to increase.

**Figure 8:**
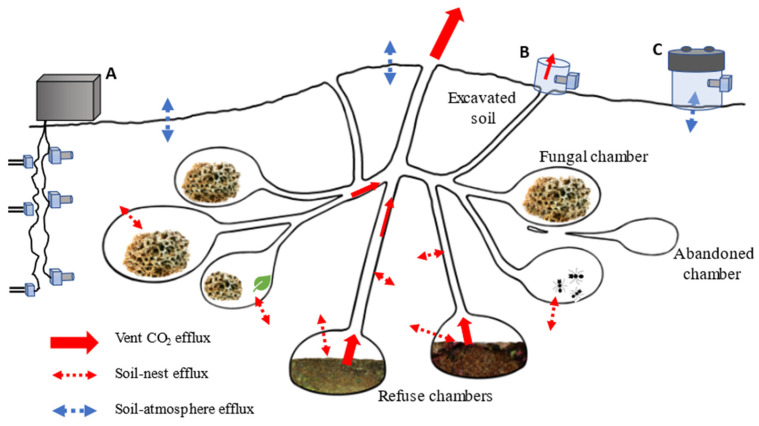
Diagram of a leaf-cutter ant (*Atta cephalotes*) nest comprised of interconnected fungal and refuse chambers. The arrows depict the CO_2_ fluxes between the nest structures, surrounding soil, and atmosphere. The sensor network components monitor: (**A**) the soil moisture, temperature, and CO_2_ concentration at multiple depths; (**B**) CO_2_ emissions from nest tunnel openings; and (**C**) the CO_2_ efflux from the soil (see text for details).

Our research investigated the carbon dynamics as influenced by leaf-cutter ants and their nests and differentiated the sources of CO_2_ efflux between the activities of ants, fungi, the nest microbial community, and roots and hyphae (Figure 8). Additionally, we quantified the nitrogen and phosphorus biogeochemistry of ant nests to determine their legacy effects after the nests are abandoned. This research involved a multifaceted approach using new technologies to couple continuous measurements of the soil CO_2_ with discrete measures of the CH_4_ efflux, as well as the isotopic δ^13^C composition, soil carbon pools and fluxes, soil nutrient concentrations, estimates of root and fungal dynamics, and microbial community and functional indices. The measurements were carried out on ant nests, paired control sites, and along a chronosequence of abandoned nests to quantify the legacy effects.

The CO_2_ concentrations in nest tunnels were higher than the background (atmospheric) levels and at times exceeded 5% (by volume) in the vents connected to the fungal and refuse chambers [42,43,44]. Thus, the nest soils adjacent to these tunnels were nutrient hot spots, emitting more CO_2_ than the soils away from the nests. By continuously excavating the ventilation network in their nests, leaf-cutter ant colonies maintain adequate CO_2_ and O_2_ concentrations (Figure 8). Prior to our research, the CO_2_ emission rates had not been well characterized, and the potential connection between the nest air and the surrounding nest soils had not been recognized (Figure 8). The CO_2_ emissions from the soil matrix to the nest air can be significant if the opposite gradient occurs and the air in the nest has a lower CO_2_ concentration than the surrounding soil. This situation can establish a significant ventilation pathway, given the large surface of the nest walls and tunnels. An accurate characterization of the nest and nest soil emissions has improved our understanding of the role of leaf-cutter ants in carbon cycling in tropical forest ecosystems. 

The traditional methods for measuring soil carbon emissions would easily overlook CO_2_ fluxes contributions from ant nests, but this blind spot could cause scientists to miss out on important emission sources. We found delicately balanced ventilation networks which leverage the gas density difference (free convection) to expel elevated CO_2_ concentrations from these large subterranean networks of tunnels, fungal gardens, and debris chambers nests [35]. At night, we observed that when the above-ground air temperature drops, warm and less dense nest air rises out of the nest. We estimated the CO_2_ emissions from the tunnels using CO_2_ sensors held over the tunnel mouths by small acrylic cylinders. The vents themselves emitted much more carbon dioxide than the soil. The measurements of the gas flowing out of individual vents showed that carbon dioxide emissions were 10,000 to 100,000 times greater than the values measured from the soil, although the vent emissions only represented a small fraction of the total emissions from the rainforest. In addition, the leaf-cutter ants themselves often responded to our disturbances by directing workers to the area to excavate new tunnels around the edges of the cylinders.

## 7. Evolving Sensor Modalities

The technologies developed for sensors to measure the temporal and spatial scales of our physical environment are generally more mature and robust in design than the existing technologies for chemical or biological sensors. A large commercial base exists for the design and manufacture of diverse modes of sensors for temperature, pressure, and physical properties (Table 1). Expanding on these existing commercial sensors, there is a need for modular sensor arrays and data loggers to provide three-dimensional parametric data for air, water, soil, and groundwater over spatial scales ranging from the micro (e.g., pore volume) to the mega (e.g., kilometer scale volumes). Innovative new imaging techniques for physical parameters are now available to assist in mapping the biogeochemical fluxes in spatial volumes at diverse scales. Multiple devices are now available to map atmospheric turbulence using 3D flow measurements at fine scales of a few cm.

There is a clear need for an innovative development of chemical sensors for use in a variety of natural and anthropogenic environments (e.g., atmospheric, terrestrial (soils and sediments), and aquatic (groundwater, fresh and marine waters). Many existing chemical sensors are not robust enough to measure the key or fundamental chemical compounds and ions in sustained operations at high acquisition rates. Moreover, there is a need for greater precision and sensitivity to expand flux measurements at dynamic spatial and temporal scales (Table 1). Many electrochemical and optical oxygen sensors are known to be prone to biofouling for short periods of time. Biological sensors would be useful to measure diverse biological reactions. Oxygen, basic nutrients, hydrogen sulfide, pH, CO_2_, NOx, SOx, CH_4_, CO, O_3_, Fe, Mn, and trace metals all may have significance in the ecosystem studies of biogeochemical fluxes and dynamics. As with the other types of sensors, the signals produced by biological sensors need to be processed in ways that allow for adaptive sensing. Furthermore, the sensors need to be capable of long-term deployment, and should be capable of integration with other sensing devices.

There is great promise in the future development and installation of new sensor networks to involve new sensor modalities and expand traditional sensors for microclimate (Table 1). These include imagers and acoustic monitoring devices as sensors for detecting motion, vocalization, and other organismal activities and behaviors. There are, nevertheless, numerous challenges in designing and deploying successful ecological sensor networks. Some of these issues relate to science-driven questions and requirements that are specific to terrestrial, soil, or aquatic domains. In an observatory mode of operation, the goal is to provide a more complete “fingerprint” of ecosystem structure and function over time and space.

Micrometeorologists have been measuring the CO_2_ and water vapor exchange between vegetation and the atmosphere since the late 1950s and early 1960s. However, the routine application of the eddy covariance methodologies and associated data management that allowed for continuous flux measurements did not occur until the 1980s. Technological advances were made at this time in sonic anemometry, infrared spectrometry, and digital computation. Further technological developments over the past several decades have facilitated a larger data storage capacity, and the improved stability and precision in instruments have enabled scientists to build on pioneering ecosystem flux studies. Today, AI (artificial intelligence) is opening the door to new ways of monitoring the pulse of our environment and addressing the issues of global change.

## 8. Challenges of Working with Sensors in a Humid Tropical Rainforest and Lessons Learned

As with any technology, there are limitations and challenges to working at La Selva with a warm and humid environment that is characteristic of tropical rainforest ecosystems. In our experience, we had to learn to anticipate practical problems when working with limited access to spare parts by developing procedures to maximize the life of the sensors and sensor arrays. Moreover, numerous modifications were necessary to successfully operate our sensor arrays under the extreme conditions of high temperatures and humidity, torrential periods of rainfall, and flooding. For example, rust and microbial growth in the humid environment requires high-quality stainless metal parts and care in the use of plastic tubing, which can be readily and quickly overgrown with fungal hyphae. The need to thoroughly seal all openings, holes, or gaps in sensor housings and instrumentation was also critically important to prevent insect colonization. We quickly learned that almost any exposed metal surface or opening on the sensors and instruments will rust, promote fungal growth, and/or be colonized by ants or other insects. Consistent AC power was generally not a problem, but power surges and brief outages are to be expected in remote field stations. AC-powered elements must be outfitted with surge protectors. Our only experience with an extended power loss at the towers occurred when thieves pulled and stole the 1 km of copper wire that linked the towers to the La Selva laboratory. 

An additional challenge of working in tropical rainforest ecosystems like La Selva is the presence of ferocious stinging ants and venomous fer-de-lance snakes. A focus on these biological hazards was a concern taken very seriously by the engineers in our group, which limited their field activities in the forest. However, we noted that the biologists in our group were much less affected.

The three original towers forming the La Selva Canopy Tower Network were erected in 2008. Since the La Selva environment had a history of strong storms with high winds and lightning, we took care to take all manner of precautions to protect the towers. The towers were carefully grounded with heavy copper cable to protect them against lightning. Intellectually, we knew that the median lifespan for tropical forest trees was about 120 years. However, we failed to appreciate that it meant that one out of 100 trees rooted close to the towers could be expected to fall each year. We were careful to cable the trees positioned near the towers to reduce potential damage from treefalls. Guywires stabilized the towers and were attached with breakaway bolts to keep the towers from being pulled down if a tree or branch were to fall on the wires.

Within six months, we had a major lightning strike to the tower that destroyed much of the instrumentation on one of the towers despite its grounding. In December 2008 and again in March 2009, we had a large tree branch fall against the guywires and damage parts of the towers, despite the special bolts, and detached the towers from their foundations. While none of the research instrumentation was seriously damaged, several projects and their data streams were interrupted, and the availability of space for projects was substantially reduced. In the following years, we experienced a large branch fall in June 2012, which seriously damaged the instrument room at the base of the towers. A treefall hit one of the towers in September 2013, and another in September 2015 knocked two towers off their foundations and pulled down the canopy walkway. A tornado in May 2018 blew down two of the towers and another tree fell against a tower. Most recently, a large falling tree in October 2021 destroyed one of the towers. 

Through all of this, the La Selva Canopy Tower Network has prevailed and continues to provide valuable data and instrumentation for a variety of research projects. We hope that the lessons we have learned will be of value to others.

## Figures and Tables

**Table 1:** Examples of major sensor modalities with comments on cost, reliability and power requirements.

Category	Example	Comments
Physical	Temperature (e.g., thermocouples, thermistors, IR sensors)	Inexpensive to intermediate cost, reliable, low powerequirements
	Relative humidity	Intermediate, reliable, low power
	Leaf wetness	Intermediate, reliable, low power
	Soil moisture	Inexpensive to moderate, issues with calibration, low power, manchoices
	PFD, total irradiance	Intermediate, reliable with calibration issues, low power
	Wind speed and direction	
	cup anemometer	Inexpensive to intermediate, reliable, fail at low wind speed, low power
	hot wire anemometer	Intermediate, less reliable, high power
	2-D/3D sonic anemometer	Intermediate to expensive, very reliable, moderate power
Chemical	Atmospheric CO_2_	Expensive, moderate power, requires careful calibration
	Soil CO_2_	Intermediate, reliable low power, requires careful calibration
	Soil CO_2_ efflux	Expensive, reliable, moderate power. Requires careful calibration
	Nitrate sensor	Expensive, under development for field use
	Phosphorus sensor	Not available for field use
Biological	Digital imagers	Moderately expensive, reliable, moderate power, high band width, software issues
	Minirhizotron camera	Expensive, variable power requirements
	Sap flow sensors	Moderate cost, reliable, calibration issues
	Acoustic sensors	Moderate, reliable, high band width, software issues

## Data Availability

Data are contained within the article.
